# Association between exposure to air pollutants and hospitalization for SARS-Cov-2: an ecological time-series study

**DOI:** 10.1590/1516-3180.2022.0210.R2.09082022

**Published:** 2022-10-03

**Authors:** Paola Cristina Ribeiro, Cristóvão José Dias da Cunha, Adriana de Oliveira Ribeiro dos Santos, Bianca Rezende Lucarevschi, Ana Cristina Gobbo César, Luiz Fernando Costa Nascimento

**Affiliations:** I MSc. Doctoral Student, Postgraduate Program on Mechanical Engineering, Department of Energy, Universidade Estadual de São Paulo (UNESP), Guaratinguetá (SP), Brazil.; II MSc. Doctoral Student, Postgraduate Program on Mechanical Engineering, Department of Energy, Universidade Estadual de São Paulo (UNESP), Guaratinguetá (SP), Brazil.; III MD, PhD. Assistant Professor, Department of Medicine, Universidade de Taubaté (UNITAU), Taubaté (SP), Brazil.; IV MD, PhD. Assistant Professor, Department of Medicine, Universidade de Taubaté (UNITAU), Taubaté (SP), Brazil.; V PhD. Assistant Professor, Instituto Federal de Educação Ciência e Tecnologia de São Paulo (IFSP), Campus Bragança Paulista (SP), Brazil.; VI MD, PhD. Researcher, Postgraduate Program on Mechanical Engineering, Universidade Estadual de São Paulo (UNESP), Guaratinguetá (SP), Brazil; and Researcher, Postgraduate Program on Environmental Sciences, Universidade de Taubaté (UNITAU), Taubaté (SP), Brazil.; Universidade de Taubaté, Postgraduate Program on Environmental Sciences, Taubaté, SP, Brazil

**Keywords:** SARS-Cov-2, Air pollutants, Nitrogen dioxide, Fine particulate matter, Costs, 2019 novel coronavirus

## Abstract

**BACKGROUND:**

Exposure to air pollutants and illness by severe acute respiratory syndrome coronavirus 2 (SARS-Cov-2) infection can cause serious pulmonary impairment.

**OBJECTIVE:**

To identify a possible association between exposure to air pollutants and hospitalizations due to SARS-Cov-2.

**DESIGN AND SETTING:**

Ecological time-series study carried out in Taubaté, Tremembé, and Pindamonhangaba in 2020 and 2021.

**METHODS:**

Study with Sars-Cov-2 hospitalizations with information on hospitalization date, sex and age of the subjects, duration of hospitalization, type of discharge, and costs of these hospitalizations. Statistical analysis was performed through a negative binomial regression, with data on pollutant concentrations, temperature, air relative humidity, and hospitalization date. Coefficients obtained by the analysis were transformed into relative risk for hospitalization, which estimated hospitalizations excess according to an increase in pollutant concentrations.

**RESULTS:**

There were 1,300 hospitalizations and 368 deaths, with a predominance of men (61.7%). These data represent an incidence rate of 250.4 per 100,000 inhabitants and 28.4% hospital lethality. Significant exposure (P value < 0.05) occurred seven days before hospital admission (lag 7) for nitrogen dioxide (NO_2_) (relative risk, RR = 1.0124) and two days before hospital admission for PM_2.5_ (RR = 1.0216). A 10 μg/m^3^ in NO_2_ concentration would decrease by 320 hospitalizations and » US $ 240,000 in costs; a 5 μg/m^3^ in PM_2.5_ concentration would decrease by 278 hospitalizations and » US $ 190,000 in costs.

**CONCLUSION:**

An association between exposure to air pollutants and hospital admission due to Sars-Cov-2 was observed with excess hospitalization and costs for the Brazilian public health system.

## INTRODUCTION

The World Health Organization (WHO) declared coronavirus disease 2019 (COVID-19) a global pandemic first detected in Wuhan, China, in December 2019.^1^ COVID-19 is a highly transmissible and fatal syndrome-induced disease, followed by severe acute respiratory disease. Typically, COVID-19 infected patients show mild to moderate symptoms, including sore throat, fever, shortness of breath, dry cough, and loss of smell and taste, while it causes pneumonia with severe acute respiratory syndrome (SARS), kidney failure, and even death in some patients.^2,3^

Initially described in December 2019 in Wuhan City, capital of China's Hubei Province, it became the center of an outbreak of pneumonia of unknown cause. In January 2020, scientists isolated a new coronavirus, severe acute respiratory syndrome coronavirus 2 (SARS-CoV-2), formerly known as 2019-nCoV.^4^

Exposure to air pollutants has been linked to hospitalizations, respiratory diseases, cardiovascular diseases, and death.^5–8^ However, studies have been carried out associating air pollution, and hospitalizations, and deaths from COVID-19; other studies have shown the interrelationship of short-term and chronic exposure to ambient air pollution and COVID-19 infection.^9,10^ Among these pollutants is nitrogen dioxide (NO_2_), a nitrogen-centered free radical mainly produced in urban areas by traffic. Ozone (O_3_) is a secondary atmospheric pollutant composed of three oxygen atoms formed at ground level by NO_2_ reactions and volatile organic compounds with sunlight.

Particulate matter is a mixture of liquid, solid, or solid and liquid particles suspended in the air and is composed of a carbonaceous core of organic compounds (polycyclic aromatic hydrocarbons, PAHs), inorganic compounds (transition metals, sulfates, and nitrates), and biological components (bacteria, fungi, and viruses). Particulate matter is categorized according to size, PM_10_, and its fine fraction PM_2.5_ with aerodynamic diameters of less than 10 and 2.5 microns, respectively.^11^

## OBJECTIVE

This study aimed to identify possible associations between exposure to air pollutants and hospitalizations due to COVID-19 in residents of the conurbation cities of Taubaté, Tremembé, and Pindamonhangaba, SP, given the fact that exposure to air pollutants is associated with diseases of the respiratory system and COVID-19 is a respiratory system's disease.

## METHODS

An ecological time-series study was carried out with data on hospitalization due to COVID-19 in residents of the conurbation cities of Taubaté, Tremembé, and Pindamonhangaba, located in Paraíba valley of São Paulo State, between April 1, 2020, and March 31, 2021.

The city of Taubaté is located between two large metropolises, Rio de Janeiro and São Paulo. With a humid subtropical climate at 580 meters above sea level, it is located in the region of the Paraíba valley and has great economic importance, predominantly industrial. With approximately 320,000 inhabitants and an area of 625 km^2^, it is considered a medium-sized city. However in the winter months, it can present peaks of pollution of fine particulate matter. This can also be attributed to the fact that the city is cut by one of the most important highways in the country, the Dutra Highway, in addition to being surrounded by the Serra do Mar and Serra da Mantiqueira, making it difficult to disperse pollutants.^12^

The city of Tremembé has about 50,000 inhabitants and an area of approximately 190 km^2^; considered a tourism resort, it has an urban area combined with the city of Taubaté; the city also has territory limits with other cities such as Pindamonhangaba, Monteiro Lobato, and Santo Antonio do Pinhal. The city of Pindamonhangaba has about 150,000 inhabitants and an approximate area of 730 km^2^. Tremembé, is also linked with Taubaté. Its economy is mainly in the service sector, followed by industry, with a humid subtropical climate at an altitude of 540 meters above sea level. Both cities are part of the metropolitan region of Vale do Paraíba.^12^

Department of Information Technology of the Unified Health System (DATASUS) provided daily values of hospitalizations,^13^ and according to the Hospital Information System of the SUS (SIHSUS) with diagnosis B34.2, which is in accordance with the ICD-10 depending on age, days of stay, date of admission, sex and type of discharge - discharge or death, and costs of admissions for both discharge and death. This SIHSUS information system has the accounting conference of hospitalizations as its main purpose. Still, it provides data such as those mentioned above that are used for studies on exposure to air pollutants and hospitalizations.

The Environmental Company of the State of São Paulo (CETESB)^14^ provided the daily values of pollutant concentrations: particulate matter with an aerodynamic diameter smaller than 2.5 u (PM_2.5_), nitrogen dioxide (NO_2_), and ozone (O_3_), in addition to data of daily temperature and relative humidity, and a correlation matrix was built with these variables.

Poisson's probability distribution is the closest to the frequency of hospitalizations since it involves discrete and counting data, with an excess of zeros and asymmetric and asymptotic distribution; however, these data may have a different mean from the variance and, for this reason, the multivariate model of negative binomial regression was used.

A multipollutant model with a confidence interval of 95% was used for the analyses, in addition to a lag period of 0 to 7 days (lag 0-7) because the effect of pollutants can be felt days after exposure. Such coefficients (coeff) were transformed into relative risk (RR), as shown in the equation: RR = exp (coeff).

In the analyses, a percentual increment (PI) of 10 μg/m^3^ in concentrations of pollutant NO_2_ and 5 μg/m^3^ in concentrations of PM_2.5_ were calculated for both sexes, represented in relative risk [RR], demonstrated in the equation PI = ([exp (ß*ΔC) −1] *100) where: ß is the value obtained from negative binomial regression, and ΔC is the variation of the pollutant concentration.

Proportional attributed risk (PAR) was used, where PAR = [1 – (1/RR)], according to PI effects in the concentration of NO_2_ and PM_2.5_, to estimate, in percentages, the impact of these increases in hospitalizations due to COVID-19. The excess of hospitalizations was calculated using the equation PAF = (PAR * N), where PAR is described above, and N is the number of hospital admissions for both sexes. The number of hospital admissions that led to death and the total cost was obtained from the DATASUS site.

The chance of death (OR) according to sex was calculated with a confidence interval of 95%. Student's t-test was used; to compare the mean age and length of stay according to sex and type of discharge – death or alive; alpha = 0.05 was the significance level adopted in this study.

The present study was not submitted to the Research Ethics Committee, as we did not have access to identifying patients during hospitalizations.

## RESULTS

A total of 1,300 cases hospitalized by CID B34.2 were identified in the three cities from April 1, 2020, to March 31, 2021. Of these hospitalizations, 742 (57.1%) correspond to males and 558 (42.9%) to females. Regarding the cases that led to death, the total was 370 (28.4%), with 229 (61.9%) corresponding to males and 141 (38.1%) to females; regarding the days of stay, according to sex, there was no statistical difference, with males having an average of 9.5 days (± 9.3) and females an average of 9.6 days (± 10.7) (P value = 0.80). The average age of admissions of adults >50 years was evident, and the average age of admissions for males was 60.2 years (± 16.6), and the average age for the female gender was 59.1 years (± 16.8), (P value = 0.23), and it can be seen in Table 1.

**Table 1:** Hospitalizations, numbers of discharges and deaths by severe acute respiratory syndrome coronavirus 2 (SARS-CoV-2), length of stay, and age, separated by sex in the conurbated cities of Taubaté, Tremembé and Pindamonhangaba, from March 2020 to April 2021

Variable	Male	Female	Total
Cases	742 (57.1%)	558 (42.9%)	**1,300 (100%)**
Deaths	229 (61.9%)	141 (38.1%)	**370 (100%)**
Length of stay (days)	9.5 (± 9.3)#	9.6 (± 10.7)#	
Age (years)	60.2 (± 16.6)#	59.1 (± 16.8)#	

# Standard deviation.

These data represent an incidence rate of 250.4 cases per 100,000 inhabitants and hospital lethality of 28.4%.

The length of stay of patients hospitalized for SARS-CoV-2 who died had an average of 12.3 (± 10.9) days, while in hospitalizations that were discharged, it was 8.5 days (± 9.3) (P value < 0.01). Analyzing hospitalizations that resulted in death, the mean age was 67.6 years (± 13.6), while the mean age of patients who were discharged was 56.8 years (± 16.6) (P value < 0.01), demonstrating that older patients are more likely to die when hospitalized.

A significant association of deaths was noted in males OR = 1.30 (95% confidence interval, CI 1.06-1.65).

The concentration of pollutants observed in the period presented an average within the standards established by the WHO, but when observing the days separately, it can be noted that the pollutant PM_2.5_ presented a maximum peak of 63 μg/m^3^, and the maximum daily value considered safe by the WHO is 25 μg/m^3^. Ozone also recorded values above WHO standards, with a peak of 110 μg/m^3^. NO_2_ remained within the acceptable standard during the study period. The pollutant values can be seen in Table 2. The values provided by Pearson's correlation matrix for the study variables are in Table 3.

**Table 2:** Values of mean, standard deviation (SD), maximum (max) and minimum (Min) of pollutants concentrations* (ug/m^3^), and meteorological variables** in the conurbated cities of Taubaté, Tremembé and Pindamonhangaba, from March 2020 to April 2021

Variable	Mean	SD	Min	Max
NO_2_	30.5	16.96	4	110
O_3_	69.2	21.07	28	131
PM_2.5_	13.9	7.35	4	63
RH (%)	43.2	14.19	16	90
MT (°C)	22.1	3.15	12.8	30.5

* NO_2_ = nitrogen dioxide; O_3_ = ozone; PM_2.5_ = fine particulate matter;

** RH = relative humidity; MT = mean temperature.

**Table 3:** Pearson's correlation matrix between all atmospheric variables, in the conurbated cities of Taubaté, Tremembé and Pindamonhangaba, from March 2020 to April 2021

	NO_2_ (ug/m^3^)	O_3_ (ug/m^3^)	PM_2.5_ (ug/m^3^)	RH (%)	Temp °C
NO_2_ (ug/m^3^)	1				
O_3_ (ug/m^3^)	0.17**	1			
PM_2.5_ (ug/m^3^)	0.51 **	0.51 **	1		
RH (%)	-0.38 **	0.60**	-029**	1	
Temp °C	-0.11**	0.55**	0.06	0.34**	1

PM_2.5_ = fine particulate matter; NO_2_ = nitrogen dioxide; O_3_ = Ozone; RH = relative humidity.

* P value < 0.05;

** P value < 0.01

**Figure 1:**
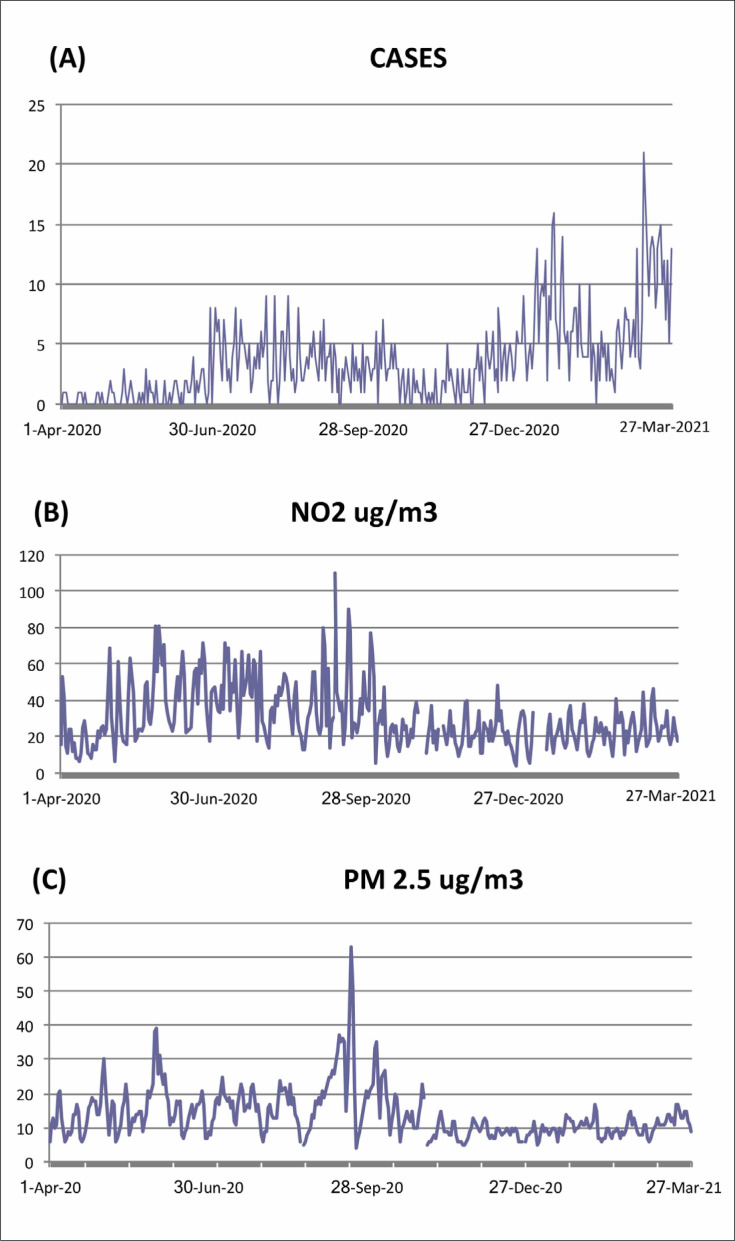
Daily values, shown in quarters, of cases (**A**), of mean concentrations of NO_2_ in ug/m^3^ (**B**) and of mean concentrations of PM_2.5_ in ug/m^3^ (**C**) in the conurbated cities of Taubaté, Tremembé and Pindamonhangaba, from April 2020 to March 2021.

The daily values of cases, as well as the values of significant concentrations of air pollutants found in the study period, are shown in Figures 1-A, 1-B, and 1-C.

When the hospitalizations that led to death were correlated with atmospheric pollutants in the multi-pollutant model, exposure to O_3_ did not show statistical significance, but exposure to NO_2_ and PM_2.5_ pollutants showed positive significance in relation to hospitalizations.

NO_2_ presented significance to the pollutant at different times with the following relative risk values and their respective confidence intervals: lag 0 [RR = 1.0108 95% CI (1.0034-1.0183)], lag 1 [RR = 1.0072 95% CI (1.0003-1.0143)], lag 3 [RR = 1.0088 95% CI (1.0018-1.0159)] and lag 7 [RR = 1.0124 95% CI (1.0051-1.0197)], whereas the pollutants PM_2.5_ showed a positive association, later when compared to NO_2_. Nevertheless, with the fine particulate matter (PM_2.5_) an association can be observed at three different times with the following relative risk values and their respective confidence intervals: lag 2 [RR = 1.0216 95% CI (1.0032-1.0403)], lag 5[RR = 1.0199 95% CI (1.0016-1.0387)] and lag 6 [RR = 1.0186 95% CI (1.0002-1.0373)].

Both hospitalizations that were discharged and hospitalizations that led to death generated costs of approximately R$ 8 million (≈ US$ 1.6 million); hospitalizations that required intensive care were responsible for 65% of the costs, and hospitalizations that resulted in death were responsible for R$ 4.5 million (≈ 51% of the total). The costs presented correspond to the hospitalizations from April 2020 to March 2021.

**Figure 2:**
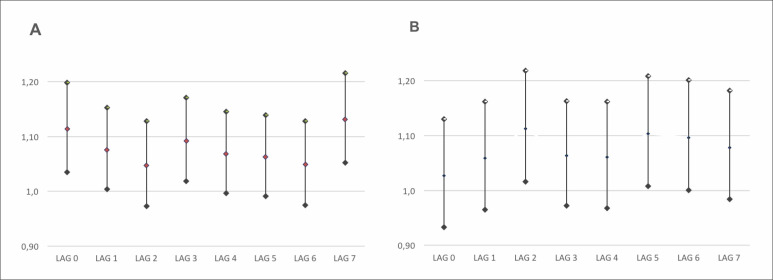
Relative risks for hospitalization due to severe acute respiratory syndrome coronavirus 2 (SARS-CoV-2), according to a 10 ug/m^3^ increase in NO_2_ (**A**) and PM_2.5_ (**B**) concentrations according to lags from 0 to 7 days in the conurbated cities of Taubaté, Tremembé and Pindamonhangaba, from April 2020 to March 2021.

Relative risk index values can be seen in Figure 2-A (NO_2_) and Figure 2-B (PM_2.5_) according to an increment of 10 μg/m^3^ in concentrations of PM_2.5_ and NO_2_.

With an increase of 10 μg/m^3^ of NO_2_ and PM_2.5_ concentrations, there would be a percentage increase of 24% and 21% in hospitalizations, corresponding to 320 and 278 hospitalizations, respectively. Thus, with the reduction in the concentrations of these pollutants, as explained above, for NO_2,_ there would be savings of around R$1.2 million (≈ US$ 240 thousand) in the cost of hospitalizations. For PM_2.5_, the reduction would reach approximately R$ 970 thousand (≈ US$ 190 thousand) in cases of hospitalization.

## DISCUSSION

This study identified hospitalization and lethality rates for SARS-Cov-2 in the conurbation cities of Taubaté, Tremembé, and Pindamonhangaba, showing a positive association with exposure to NO_2_ and PM_2.5_, while exposure to O_3_ showed a non-significant association; such data provide a basis for further studies to be carried out in other regions, especially the heavily polluted ones.

Effects were noted at lags 0, 1, 3, and 7 for exposure to NO_2_ and lag 2, 5, and 6 for PM_2.5_.

The data obtained represent an incidence rate of 250.4 cases per 100,000 inhabitants and a case fatality rate of 28.4%, with both hospitalizations and deaths predominating in males.

A study carried out with data of the SIVEP-Gripe official system of the Ministry of Health, obtained between February 2020 and May 2021, identified 366,802 cases and 106,437 deaths for the entire state of São Paulo. This indicates an accumulated incidence of 858.6 cases per 100,000 inhabitants and a mortality rate of 259.1 per 100,000 inhabitants; these values, well above those found in our study, may be associated with geographic differences and differences in the source of data collection. Males had a higher prevalence of hospitalizations and deaths than females, which was similar to the findings of our study.^15^ The same behavior was found by Peres et al.^16^ and Klokner et al.^17^

This relatively unequal incidence and mortality in men can be interpreted considering many factors: the comparatively higher prevalence of comorbidities (hypertension, diabetes, cardiovascular diseases, and chronic lung diseases), more risk behaviors (smoking and alcohol use), and exposure to occupational and sex differences in immune responses.^18,19^

In a study carried out in India, where the concentrations of PM_2.5_ were 88.3 ug/m^3^ and those of NO_2_, 36.5 ug/m^3^, associations were also identified between exposure to these pollutants and new cases of COVID-19 as well as an association with new deaths.^20^

Daily confirmed cases in 120 Chinese cities were obtained from January 23, 2020, to February 29, 2020, where significantly positive associations were observed for PM_2.5_ and NO_2_ exposure. A 10 μg/m^3^ (lag 0-14) increase in PM_2.5_ and NO_2_ was associated with 2.24% (95% CI: 1.02 to 3.46) and 6.94% (95% CI: 2.38 to 11.51) in the daily count of confirmed cases, respectively.^21^

In two regions of northern Italy, with tropospheric nitrogen data estimated by satellite, even with low model accuracy, it was possible to identify an association between high concentrations of NO_2_ and deaths from COVID-19, which provides evidence supporting a pollution effect in increasing the proportion of fatal cases of the disease. The association was stronger when using the longer-term cumulative mortality as an outcome.^22^

Another important data revealed in this study is the cost of these hospitalizations. Hospitalizations that resulted in hospital discharge cost R$ 3,526,328.67, and the hospital cost for patients who died cost R$ 4,538,663.57; hospitalizations that resulted in death and that required intensive care (ICU) cost twice as much as those that resulted in ICU discharge.

If they reduced 5 μg/m^3^ of the pollutant PM_2.5_ in the atmosphere and 10 μg/m^3^ of the pollutant NO_2_, in the case of the studied region, the savings could be up to R$ 1.2 million.

The mechanisms involved are still poorly understood. It is believed that increased oxidative stress is the key mechanism of pollutant-induced toxicity and that PM_2.5_ suspended in the atmosphere would facilitate viral survival and promote its atmospheric transport. Exposure to air pollutants promotes viral entry, replication, and assembly, which cause increased local inflammation due to reduced mucociliary clearance, modulation of cellular pathways, and increased epithelial permeability because of decreased junction proteins with a substantial increase in viral spread and inflammation due to permeable epithelium, prevention of macrophage uptake and defects in natural killer (NK) cell functions with amplification of inflammation and neutrophil recruitment plus increased virus-induced tissue damage and inflammation. This sequence of events leads to fluid accumulation in the alveoli, respiratory failure, and death.^23^

This study had limitations. First, due to ecological studies, the type of information obtained from an official source might have a diagnostic error. Second, the address of the subject who informed it possibly wrongly. Third, lack of information on co-morbidities might have contributed to the impossibility of assessing the importance of risk factors mentioned in the literature and estimating their importance in the number of cases. Moreover, exposure to pollutants might not be indicated as a cause of infection by COVID-19, but an association between exposure and cases.

## CONCLUSION

Regardless of what might have caused the possible abovementioned limitations, it was possible to identify an association between exposure to PM_2.5_ and NO_2_ pollutants in hospitalizations due to COVID-19, in addition to the total cost of these hospitalizations.
